# New Local Anesthetic

**Published:** 1903-03-15

**Authors:** 


### NEW LOCAL ANESTHETIC.

Paramidobenzoic acid ether (anesthetic of Ritsert), a non-poisonous substitute for cocain. By Dr. Schaeffer-Stuckert, Frankfort-on-the-Main.

The author states the common desire for a local anesthetic possessing the efficacy of cocain without its toxic quality, and says that Dr. Ritsert has come near the fulfilment of this desire in the production of the ethyl-ether of paramidobenzoic acid, the formula of which is $C_6H_4<^{NH_2}_{COOC_2H_5}$. This compound he produced in 1890 and recognized as a local anesthetic. Owing to its insolubility in water, however, for some time no practical use was made of the drug. Later, interest in it was revived, and a number of prominent medical men made use of it with success. The author was requested by Dr. Ritsert to determine its value in dental practice.

The drug is described as a white, odorless powder, soluble with difficulty in water, easily soluble in alcohol, ether, chloroform, acetone, also in fats and oils. The authors first experiments were made with an oily solution, though this was not satisfactory for subcutaneous injections. Later, Dr. Ritser found a way to make watery solutions, and a one-quarter to one percent solution was used in subsequent experiments. Sundry experiments upon animals conducted by reliable people are mentioned to prove the non-toxic quality of the drug. Small and medium doses produced no harmful effect, and enormous doses only a slight effect, which resembled that of phenacetin, to which it is chemically allied.

The author's investigations have been directed toward proving the usefulness of the drug for the following purposes: 1. For subcutaneous injection in the extraction of teeth; 2. For the painless destruction of the pulp in combination with arsenic; 3. For the treatment of pain in tooth-sockets and wounds.

For the purpose of tooth extraction, one cubic centimeter of a one-percent solution is used, and the author states that the results are very satisfactory.

For the destruction of pulps, a mixture of equal parts of arsenic and the anesthetic in question, combined with oil of cloves, is used. This paste, when used under Fletcher's cement, or any other covering which is devoid of pressure, produces, inside of twenty-four hours a painless destruction of the pulp.

In the treatment of pain in tooth-sockets and wounds the author applies the drug in powder to the affected surfaces, sometimes mixing it with dermatol, and finds the greatest satisfaction in such use.

This preparation is sold by Apotheker Weinreben, Frankfort-on-the-Main, Germany.—*International.*

